# Hydatids of the Lower Lip

**Published:** 1847-09

**Authors:** 


*Hydatids of the Lower Lip.*—Dr. Heller, of Stuttgard, has seen five cases in which acephalo-cysts were developed in the lower lip, and always on its inside. The causes of this affection are unknown; in two cases it was attributed by their patients to their having bitten the lip. It appears first as a small and hard lump, which rapidly increases, so that in one month it may attain the size of a cherry; it gives rise to pain, deformity, and difficulty in moving the lip. The hydatids are seated immediately beneath the mucous membrane, and are transparent. The largest Dr. Heller has seen was of the size of a nut. If one succeeds in removing them without opening their cavity, they are found to be made up of a round vesicle, full of fluid, transparent as water, and with a diaphanous and tender wall. The only means of curing this affection consists in the extirpation of the cyst, and this must be total, otherwise it will reappear; hence, as a precautionary measure, nitrate of silver may be applied after the removal of the tumour.—*Ibid.*

